# Supplementary figures and images for: Detection of single nucleotide polymorphisms in virus genomes assembled from high-throughput sequencing data: large-scale performance testing of sequence analysis strategies
Source: PeerJ. 2023 Aug 16;11:e15816. doi: 10.7717/peerj.15816 (PMC10439718; doi:10.7717/peerj.15816)

Sample 1 R1


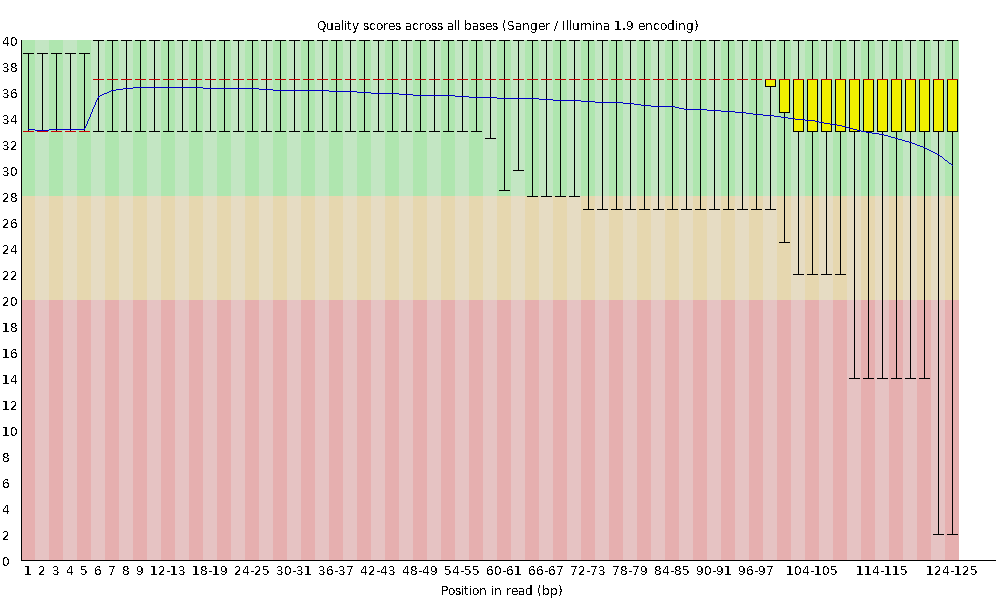


Sample 1 R2


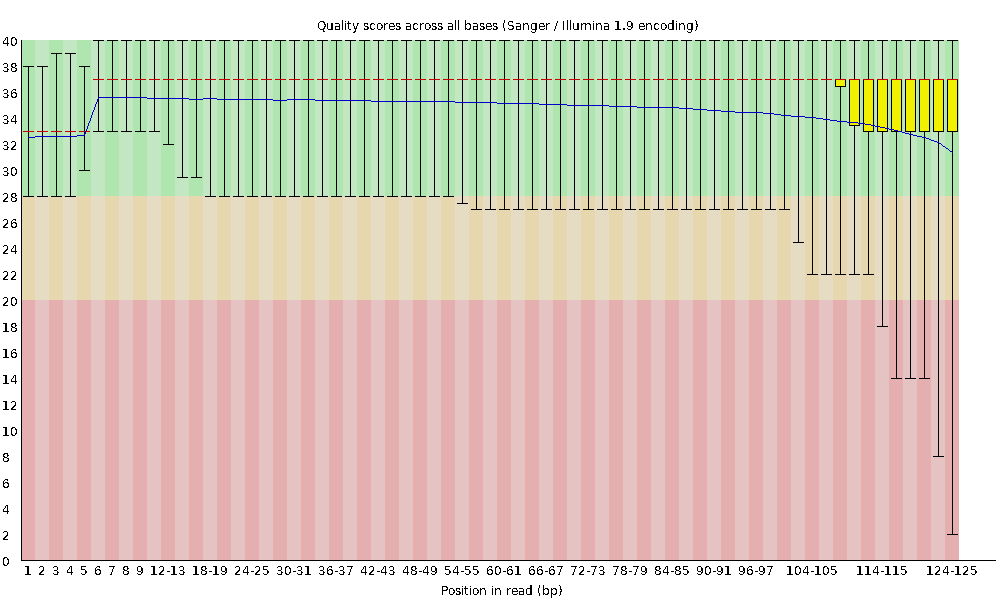


Sample 2 R1


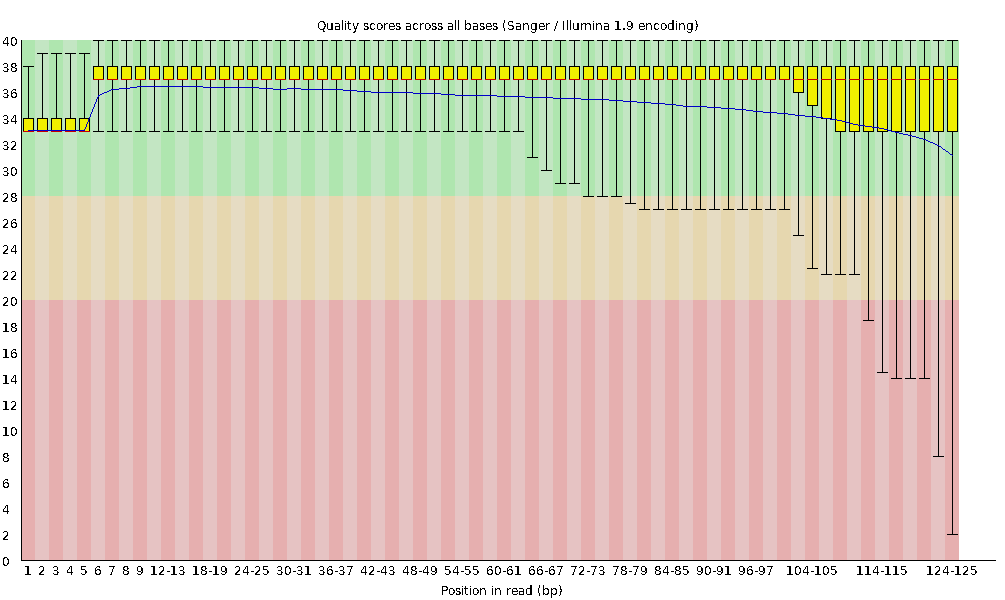


Sample 2 R2


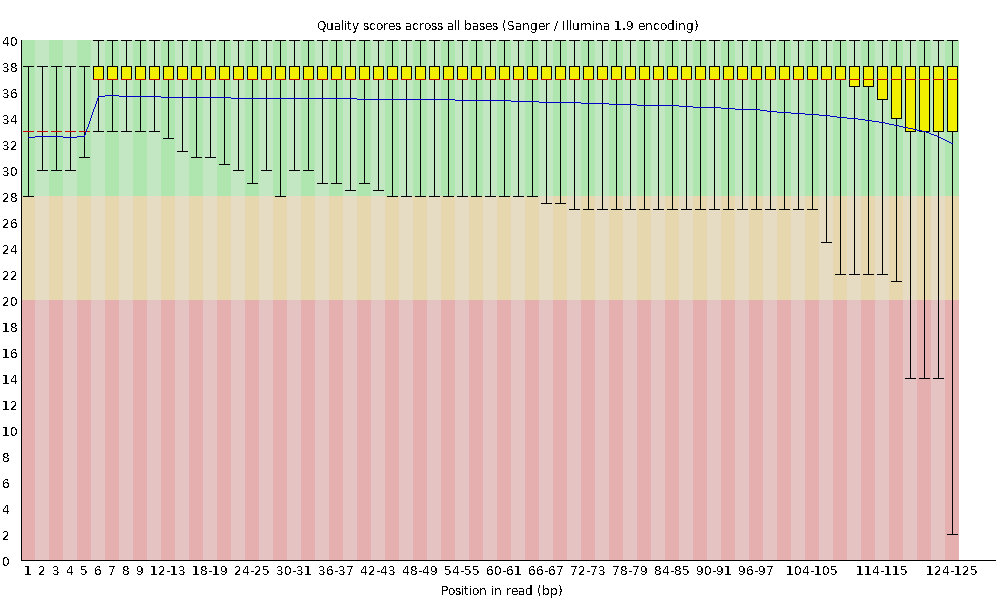


Sample 3 R1


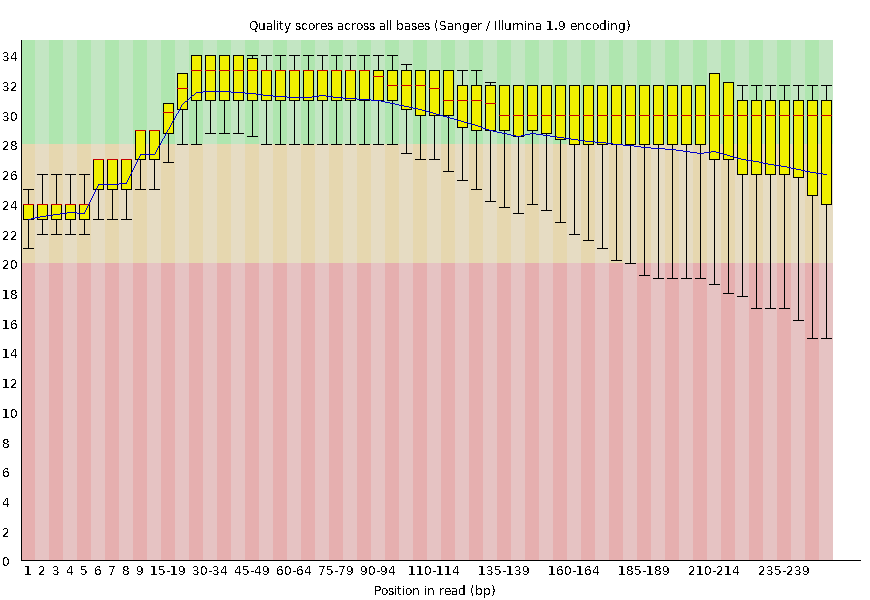


Sample 3 R2
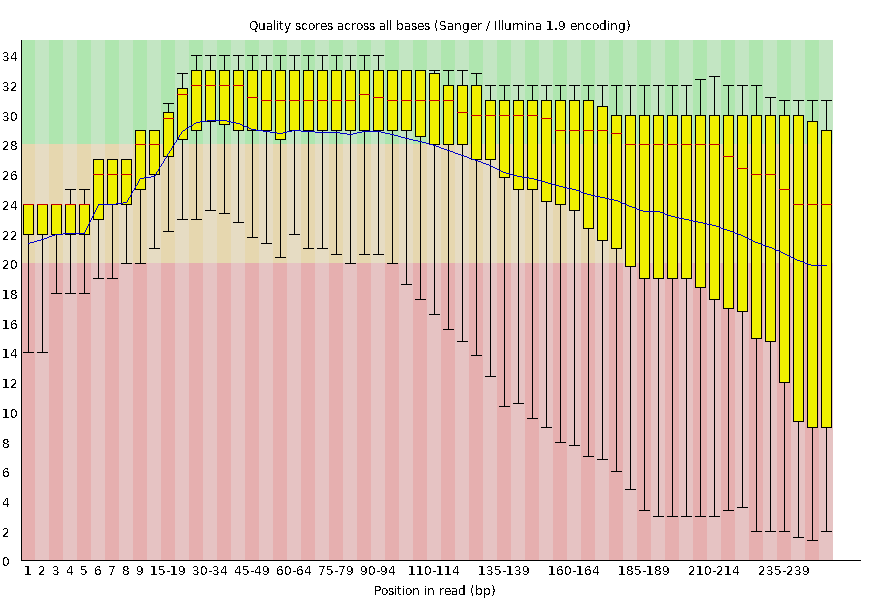

Supplement: Supplemental Information 1 [file peerj-11-15816-s001.docx]
